# DIlp7-Producing Neurons Regulate Insulin-Producing Cells in *Drosophila*

**DOI:** 10.3389/fphys.2021.630390

**Published:** 2021-07-27

**Authors:** Elodie Prince, Jenny Kretzschmar, Laura C. Trautenberg, Susanne Broschk, Marko Brankatschk

**Affiliations:** ^1^Biotechnologisches Zentrum, Dresden, Germany; ^2^CNRS UMR 7277, Inserm U1091, UNS – Bâtiment Centre de Biochimie, Faculté des Sciences, iBV – Institut de Biologie Valrose, Nice, France; ^3^Applied Zoology, Faculty of Biology, Technische Universität Dresden, Dresden, Germany

**Keywords:** insulin signaling, AKT, metabolism, heat resistance, diet

## Abstract

Cellular Insulin signaling shows a remarkable high molecular and functional conservation. Insulin-producing cells respond directly to nutritional cues in circulation and receive modulatory input from connected neuronal networks. Neuronal control integrates a wide range of variables including dietary change or environmental temperature. Although it is shown that neuronal input is sufficient to regulate Insulin-producing cells, the physiological relevance of this network remains elusive. In *Drosophila melanogaster*, Insulin-like peptide7-producing neurons are wired with Insulin-producing cells. We found that the former cells regulate the latter to facilitate larval development at high temperatures, and to regulate systemic Insulin signaling in adults feeding on calorie-rich food lacking dietary yeast. Our results demonstrate a role for neuronal innervation of Insulin-producing cells important for fruit flies to survive unfavorable environmental conditions.

## Author Summary

How can fruit flies roam ecological systems in the northern hemisphere? Seasonal temperature changes and daily fluctuations force flies to acclimatize rapidly. In order to survive heat insects need to scale their metabolic rate. To accelerate turnover, *Drosophila* rely on different metabolic circuits including Insulin signaling. It was shown that dietary lipids originating from commercial yeast are capable to promote metabolism. However, similar optimal food sources are patchy in nature and exist only for limited time periods. Thus, adult wild flies migrate and need to feed occacionaly on alternative diets. In contrast, larvae are restricted in their mobility and need to live from local resources. We found that the conserved Insulin-like peptide7 (dIlp7) produced by dIlp7-neurons is critical to maintain basic Insulin signaling levels of adult flies feeding on calory-rich diets lacking yeast lipids. In larval development, evidence from genetic interactions position dIlp7 upstream of dIlp2. DIlp2 is produced by Insulin-producing cells and we propose that dIlp7 inhibits its activity in larvae kept on yeast-free food. Interestingly, genetic interactions mapped in larval development do not seem to play a role in adult *Drosophila* and in flies the absence of dIlp7 lowers Insulin signaling levels in general. Taken together, Insulin-dependent metabolism in flies living on food sources lacking yeast is regulated by neuronal dIlp7 responsible to maintain basic Insulin signaling levels. We propose that dIlp7 is critical for wild flies to survive on plant diets, poor in microbes.

## Introduction

Invading new ecological systems, *Drosophila* adapted to local food sources (Robertson, 1966; Koyama et al., 2020). Such resources can vary their composition in response to environmental temperature changes (Carvalho et al., 2012; Brankatschk et al., 2018; Knittelfelder et al., 2020). Wild *Drosophila melanogaster* prefer to feed on rotting fruits, which represent a diet composed of microbes and plant material. As such food sources are erratic, migrating flies need to feed on alternative carbohydrate sources, such as nectar. such as nectar. Nectar represents a type of calorie-rich but microbe-poor plant food (Good and Tatar, 2001). In addition, seasonal and diurnal temperature fluctations can reach extremes (Brankatschk et al., 2018). Whereas, more mobile adult flies are able to avoid unfavorable weather by hiding in suitable places (Garrity et al., 2010; Vijendravarma et al., 2012; Brankatschk et al., 2018), developing less mobile larvae have to nourish on given diets and to endure environmental hardships.

We have shown that *Drosophila* larvae struggle to survive on experimental yeast-free plant diets at high temperatures; however, these food types support development in cold conditions (Brankatschk et al., 2014, 2018). To survive heat stress, larvae need to accelerate their metabolism. By feeding on yeast, *Drosophila* maintain high Insulin Signaling (IS) levels required to shuttle efficiently nutritional cues, such as circulating sugars, into cells (Ikeya et al., 2002; Brankatschk et al., 2014; Graham and Pick, 2017; Volkenhoff et al., 2018; Hertenstein et al., 2020). *Drosophila* produces eight different Insulin-like peptides (dIlps). The dIlp structure is conserved and thus, similar to human Insulin or Insulin-like growth factor (IGF) peptides (Brogiolo et al., 2001; Rulifson et al., 2002; Grönke et al., 2010; Veenstra, 2020). However, individual dIlp peptides can have very different roles in larval development compared to adult lifehood. Some dIlps play exclusive developmental roles, such as dIlp1 (Slaidina et al., 2009; Liu et al., 2016; Liao et al., 2020), dIlp 4 (Brogiolo et al., 2001) and dIlp8 (Colombani et al., 2003, 2012, 2015; Garelli et al., 2015), and their production is restricted in time. The other dilps are important throughout the life of fruit flies, but can have specialized functions (Chell and Brand, 2010; Bai et al., 2012; Kubrak et al., 2014; Spéder and Brand, 2014; Schiesari et al., 2016). Some dIlps are produced in neurons, such as Insulin-producing cells (IPCs, namenly dIlp 2,3,5) or dIlp7-producing neurons (D7Ns). Neuronal dIlps are secreted into circulation (Rulifson et al., 2002; Miguel-Aliaga et al., 2008; Cognigni et al., 2011; Nässel et al., 2013), but all dIlp-producing cells have additional targets in the brain (Nässel and Homberg, 2006). D7Ns are wired with IPCs in larval and adult brains, and are able to stimulate IPC related dIlp production (Nässel et al., 2013). However, the function of their main product dIlp7 remains unclear (Krieger et al., 2004; Riehle et al., 2006; Grönke et al., 2010).

Here, we investigate IS in the scope of different diets and environmental temperatures. We show that the activity of D7Ns is important to protect larvae from heat stress and that dIlp7 regulates dIlp2 activity to control larval development on yeast-free diets. In addition, we found that adult flies kept on yeast-free plant food require dIlp7 to maintain systemic IS. We show evidence that dIlp7 targets the leucin-rich repeat-containing G protein-coupled receptor 3 (Lgr3) expressed by IPCs and that yeast products can supplement for the loss of dIlp7. Taken together, we propose that D7Ns form a neurosecretory network essential to sustain *Drosophila* in absence of dietary yeast and changing environmental conditions.

## Results

### DIlp7-Producing Neurons Promote Heat Resistance to *Drosophila* Larvae

Previous studies demonstrated the importance of dietary yeast for an efficient high temperature response (Brankatschk et al., 2018). However, it remains unclear if yeast have additional roles than to facilitate simply dIlp secretion by IPC neurons (Brankatschk et al., 2014). To investigate the interplay of yeast and IPCs, we first tested the necessity of IPC activity for larval survival at 28°C on yeast food (YF, Table 1). To do so, we silenced IPCs by expressing the inward rectifier potassium channel Kir2.1 (MacLean et al., 2002; Brankatschk et al., 2014) at different developmental stages. We found that IPC activity is essential for each larval developmental stage (Supplementary Figure 1A). On yeast-free diets, IPC activity is low and the secretion of dIlp peptides strongly reduced, and larval survival poor (Brankatschk et al., 2014, 2018). To ask whether high IPC activity in yeast-free environmens is sufficient to provide heat resistance, we kept TRPA1 (thermo-sensitive transient receptor potential cation channel A1)-expressing larvae on corn food (CF, Table 2) and induced high Ca^2+^ levels in these cells by a temperature shift to 28°C. However, high cellular Ca^2+^ levels did not improve larval development indicating that yeast products regulate also cell types other than IPCs (Supplementary Figure 1B).

**TABLE 1:** Yeast food recipe.

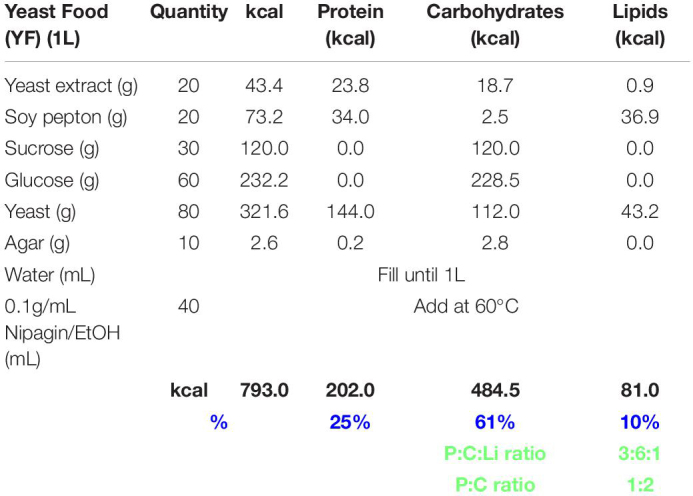

*Recipe for 1liter of yeast food. The calories (in kcal) brought by each ingredients have been calculated. At the bottom, in black bold, the totals (in kcal) aer shown. In blue are indicated the proportions (%) of kcal of proteins, carbohydrates, or lipids on the total amount of calories. In green are shown the protein: carbohydrate: lipid and protein: carbohydrate ratios. EtOH, ethanol; P, proteins; C, carbohydrates; Li, lipids.*

**TABLE 2:** Corn food recipe.

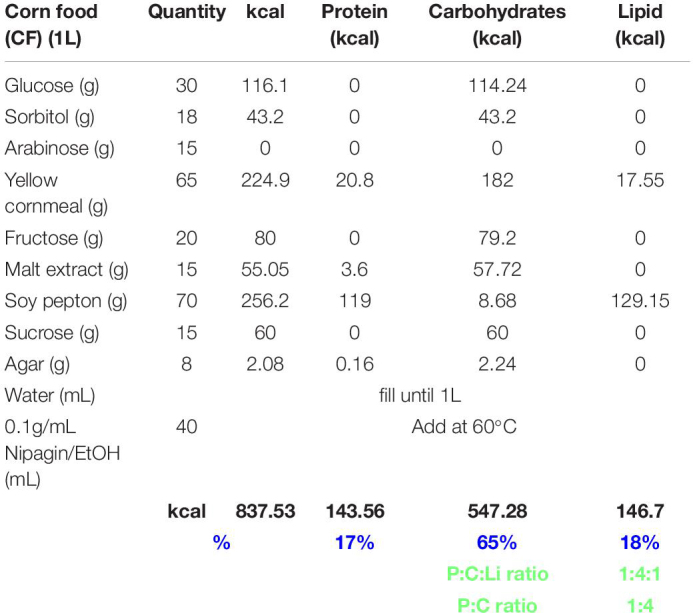

*Recipe for 1liter of corn food. The calories (in kcal) brought by each ingredients have been calculated. At the bottom, in black bold, the totals (in kcal) aer shown. In blue are indicated the proportions (%) of kcal of proteins, carbohydrates, or lipids on the total amount of calories. In green are shown the protein: carbohydrate: lipid and protein: carbohydrate ratios. EtOH, ethanol; P, proteins; C, carbohydrates; Li, lipids.*

**FIGURE 1:**
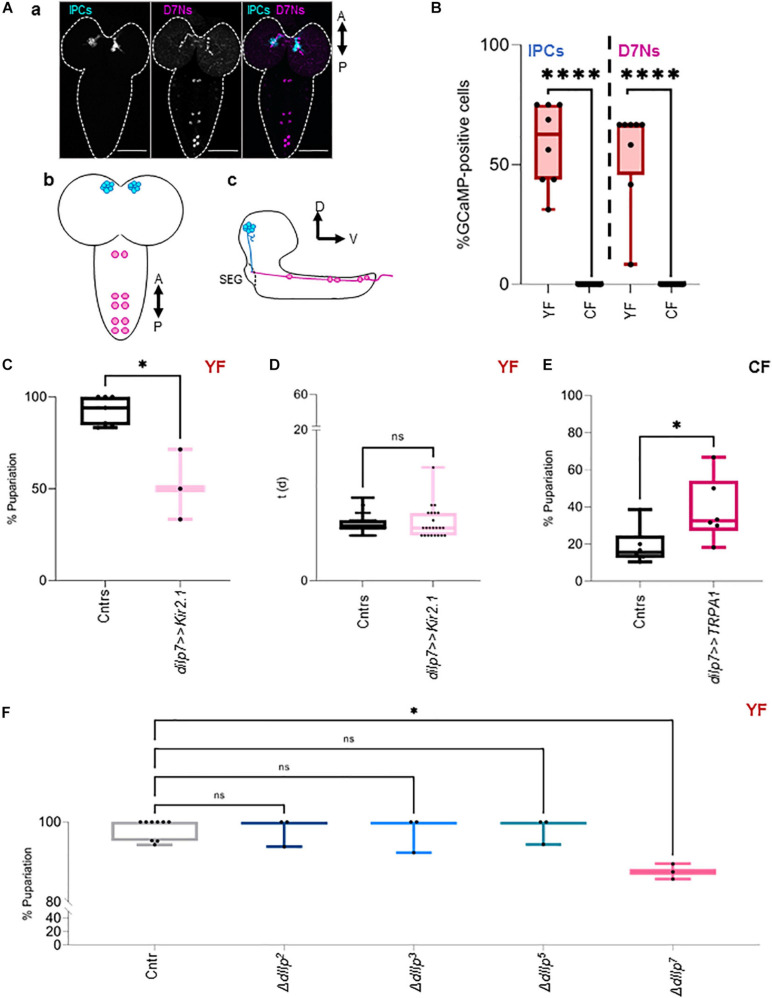
DIlp7-producing neurons provide heat resistance. **(A)** Confocal Z-projection (a, scale bar = 50 μm) and graphical depictions (b,c) of larval brains. Shown are the relative position of Insulin-producing cells (IPCs, blue) and dIlp7-neurons (D7Ns, magenta). Crossed arrows point out relative view axis. **(B)** Fluorescent GCaMP neurons were counted in fixed brains from larvae kept on yeast food (YF, red box-plots) or corn food (CF, black box-plots) at 20°C. Shown are the percentage of activated cells for n brains (black dots). Significance calculated by Mann-Whitney’s test, *p* < 0.0001^****^. For crude data, see Supporting informations_Raw data_Main figures/Supplementary Figure 1B (C,D) Plotted are the percentage of formed pupae **(C)** or the time (in days, d) to reach pupariation **(D)** of individual larvae kept on yeast food (YF). Compared are genetic controls (Cntrs; pooled data of *dilp7-Gal4/+* and *UAS-Kir2.1/+*) and larvae expressing Kir2.1 in D7Ns (*dilp7 > > Kir2.1*, light-pink) at 28°C. **(C)** Box plot, each black dot represents one experiment with minimum *n* = 6 individual larvae. Significance calculated by Mann-Whitney’s test, *p* = 0.0167^∗^. For crude data, see Supporting informations_Raw data_Main figures/Supplementary Figure 1C. (D) Box-plot, each black dot represents one larva. Significance calculated by Kruskal-Wallis test, *p* = 0.7457^*ns*^. For crude data, see Supporting informations_Raw data_Main figures/Supplementary Figure 1D. (E) Plotted are the percentage of formed pupae of genetic controls (Cntrs; pooled data of *dilp7-Gal4/+* and *UAS-TRPA1/+*) and larvae expressing TRPA1 in D7Ns (*dilp7 > > TRPA1*, dark-pink) kept on corn food (CF) at 28°C. Box-plot, each black dot represents one experiment with minimum *n* = 6 individual larvae. Significance calculated by Mann-Whitney’s test, *p* = 0.0411^∗^. For crude data, see Supporting informations_Raw data_Main figures/Supplementary Figure 1E. (F) Shown is the developmental success on yeast food (YF) at 28°C. Plotted are the percentage of formed pupae of genetic controls (*mCherry:FOXO*, Cntr) and different dIlp mutants as indicated on the *X*-axis. Box plot, each dot represents one experiment with minimum *n* = 6 individual larvae. Statistics, Kruskal-Wallis test, *p* > 0.9999^*ns*^; *p* = 0.0141^∗^. For crude data, see Supporting informations_Raw data_Main figures/Supplementary Figure 1F.

D7Ns produce dIlp7 and are wired with IPCs in larval and adult stages [Figure 1A and Miguel-Aliaga et al. (2008), Cognigni et al. (2011), Nässel et al. (2013)]. It was shown that these neurons and IPCs increase intracellular Ca^2+^ levels in response to dietary yeast (Brankatschk et al., 2014; Linneweber et al., 2014). To assess Ca^2+^ levels of both neuronal subsets in larvae kept on CF or YF, we expressed the fluorescent Ca^2+^ reporter GCaMP (Brankatschk et al., 2014; Linneweber et al., 2014) and analyzed the state of the fluorophore directly on fixed samples (Brankatschk et al., 2014). We found that our approach was unable to detect measurable Ca^2+^ levels in IPCs and D7Ns on CF contrary to neurons from larvae kept on YF (Figure 1B and Supplementary Figure 1C). To test if D7N activity changes heat response, we expressed Kir2.1 (*dIlp7 > > Kir2.1*) and tracked the larval development on YF at 28°C. We found that low D7N activity results in reduced larval survival (Figure 1C); however, the developmental speed of surviving animals remains comparable to genetic controls (*dIlp7-Gal4/+* and *UAS-Kir2.1/+*; Figure 1D). To test if D7N activity is sufficient to provide heat resistance, we expressed TRPA1 (*dIlp7 > > TRPA1*), and tracked the pupariation of larvae kept on CF. Of note, rapid temperature changes in TRPA1 expresing neurons could induce thermonociceptive sensation manifesting behavior like larval rolling. We did not observe such stress reactions and presume that D7Ns are not part of a TRPA1 dependent nociceptory network (Hu et al., 2017). We show that survival rates of TRPA1-expressing larvae are significantly higher with respect to genetic controls (*dIlp7-Gal4/+* and *UAS-TRPA1/+*; Figure 1E). The Insulin peptide, dIlp7, is one main product of D7Ns. To investigate if dIlp7 contributes to heat resistance, we tracked the development of Δ*dIlp^7^* mutants at 20°C and 28°C (Figure 1F and Supplementary Figure 1D). We found that Δ*dIlp^7^* kept on YF show no or only some temperature sensitivity with respect to controls (*mCherry:Foxo*, Cntr) and mutants deficient in the production of either dIlp2, dilp3 or dIlp5 (Figure 1D).

Taken together, dietary yeast activate IPCs and D7Ns and both cell types are essential for larvae to survive heat stress. Whereas high Ca^2+^ levels in D7Ns are sufficient to partially rescue survival on yeast-free CF at high temperatures, high Ca^2+^ levels in IPCs are ineffective. Thus, we speculate that D7Ns modulate the secretion of particular IPC dIlp peptides to maximize the efficiency of the IS cascade.

### On Yeast-Free Food, dIlp2, and dIlp7 Are Essential for Larval Development

**FIGURE 2:**
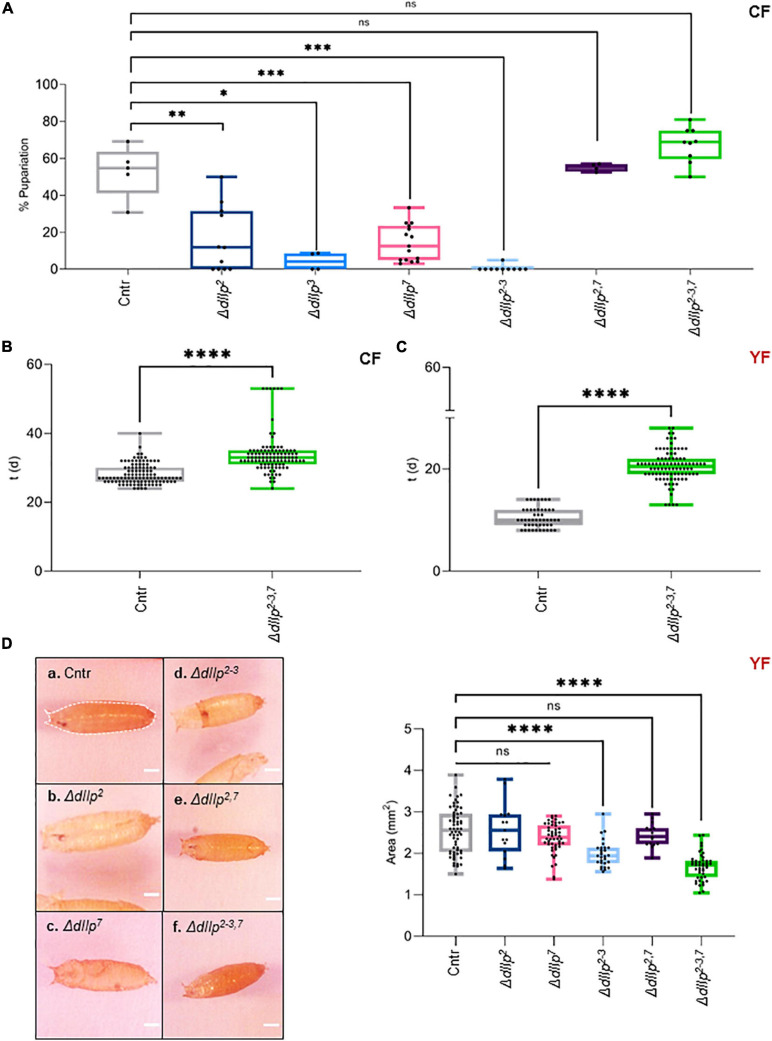
DIlp7 regulates larval development on yeast-free corn food. **(A)** Plotted is the larval survival on corn food (CF) at 20°C. Shown are the percentage of formed pupae from genetic controls (*mCherry:FOXO*, Cntr) and different dIlp mutants as indicated on the *X*-axis. Box-plot, each dot represents one experiment with minimum *n* = 10 individual larvae. Significance calculated by Mann-Whitney’s test, Cntr vs. Δ*dIlp^2^*: *p* = 0.0025^∗∗^; Cntr vs. Δ*dIlp^3^*: *p* = 0.0159^∗^; Cntr vs. Δ*dIlp^7^*: *p* = 0.0002^∗∗∗^; Cntr vs. Δ*dIlp^2–3^*: *p* = 0.0003^∗∗∗^; Cntr vs. Δ*dIlp^2,7^*: *p* > 0.9999^*ns*^; Cntr vs. Δ*dIlp^2–3,7^*: *p* = 0.0889^*ns*^. For crude data, see Supporting informations_Raw data_Main figures/Supplementary Figure 2A. (B,C) Shown as box-plots are the larval developmental speed (in days, d) on corn food (CF, **B**) or yeast food (YF, **C**) at 20°C. Compared are genetic controls (*mCherry:FOXO*, Cntr) with Δ*dIlp^2–3,7^* triple mutants. For crude data, see Supporting informations_Raw data_Main figures/Supplementary Figures 2B,C. Significance calculated by Mann-Whitney’s test, *p* < 0.0001^****^. **(D)** Shown are photographs (a–f) of pupae formed from larvae kept on yeast food (YF) at 20°C. Pupal areas (indicated in a., broken line) of genetic controls (*mCherry:FOXO*, Cntr) and different dIlp mutants were measured and plotted on a box-plot (one black dot = one pupa). Significance calculated by Mann-Whitney’s test, Cntr vs. Δ*dIlp^2^*: *p* = 0.9366^*ns*^; Cntr vs. Δ*dIlp^7^*: *p* = 0.1017^*ns*^; Cntr vs. Δ*dIlp^2–3^*, Δ*dIlp^2–3,7^*: *p* < 0.0001^****^; Cntr vs. Δ*dIlp^2,7^*: *p* = 0.5218^*ns*^. For crude data, see Supporting informations_Raw data_Main figures/Supplementary Figure 2D.

We found that activated D7Ns promote survival on yeast-free CF. To investigate the role of dIlps in animals kept on CF, we performed a metabolic screen of Δ*dIlp* mutants on CF. We found that the neuronal dIlps 2, 3, and 7 are essential to promote survival and development (Figures 2A,B and Supplementary Figure 2A). To test for redundancy between identified dIlp candidates, we have used Δ*dIlp^2,3^* and Δ*dIlp^2–3,7^* mutants. Whereas Δ*dIlp^2,3^* do not pupariate on CF, Δ*dIlp^2–3,7^* larvae show developmental success rates comparable to genetic controls (Figure 2A). To evaluate potential genetic interactions between dIlp7 and each of these candidates, we have created Δ*dIlp^2,7^*, Δ*dIlp^3,7^*, and Δ*dIlp^5,7^* double mutants. Remarkably, the absence of dIlp2 and dIlp7 restored survival alike Δ*dIlp^2–3,7^*. In contrast, Δ*dIlp^3,7^* or Δ*dIlp^5,7^* animals did not perform better than respective individual mutants (Figure 2A and Supplementary Figure 2A). To test if dietary yeast compensate the loss of dIlp7, we repeated the metabolic screen using YF (no dietary plant components, Table1). Whereas most single and double mutants did not show significant phenotypes (Supplementary Figure 2B), the developmental speed of Δ*dIlp^2–3,7^* was slow (Figure 2C) and forming pupae appeared small in size with respect to controls (Figure 2D and Supplementary Figure 2C). Our data show that dIlp7 is critical for larval development on yeast-free diets. In addition, tested genetic interactions suggest dIlp7 as a regulator for the activity of dIlp2.

### Dilp7 Is Essential for Adults to Thrive on Yeast-Free Diets

**FIGURE 3:**
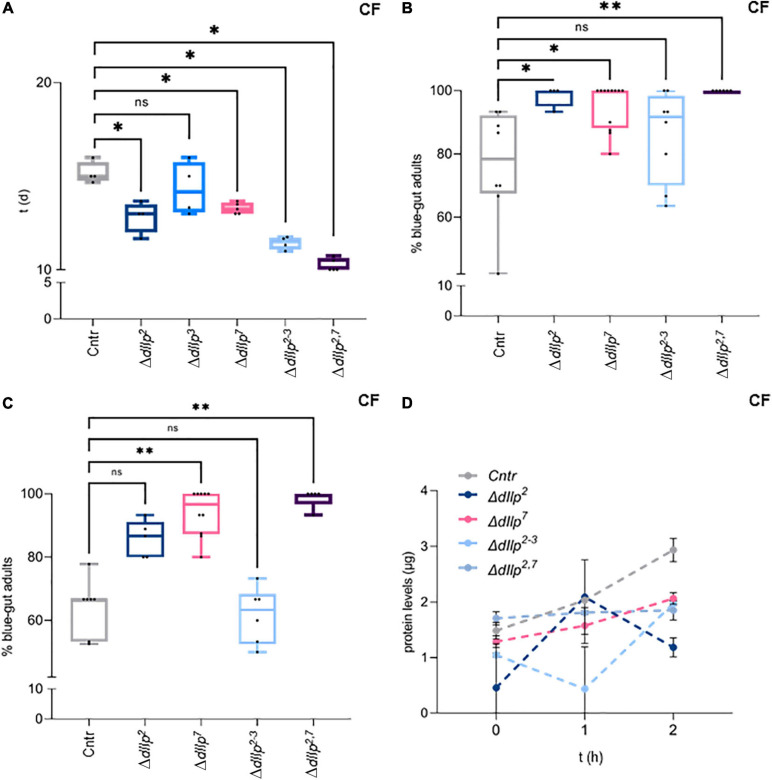
DIlp7 regulates food intake, transition and absorption of adult flies kept on yeast-free food. **(A)** Shown are box-plots depicting survival rates of staged adult flies transferred onto yeast-free corn food (CF) at 25°C. Plotted is time (in days, d) to reach 50% lethality for genetic controls (*mCherry:FOXO*, Cntr) and different dIlp mutants as indicated. One black dot = one experiment. Significance calculated by Mann-Whitney’s test, Cntr vs. Δ*dIlp^2^*, Δ*dIlp^2–3^*: *p* = 0.0286^∗^; Cntr vs. Δ*dIlp^3^*: *p* = 0.5714^*ns*^; Cntr vs. Δ*dIlp^7^*, Δ*dIlp^2,7^*: *p* = 0.0159^∗^. For crude data, see Supporting informations_Raw data_Main figures/Supplementary Figure 3A. (B,C) Plotted are the percentage of adult females kept on dye-containing corn food (CF) with colored abdomens after 4 h **(B)**, and colored-abdomen flies transferred onto dye-less corn food (CF) with colored abdomens after 4 h (C) at 20°C. The data for genetic controls (*mCherry:FOXO*, Cntr) and different dIlp mutants are depicted as box plots; one black dot = one experiment. (B) Significance calculated by Kruskal-Wallis test, Cntr vs. Δ*dIlp^2^*: *p* = 0.0282^∗^; Cntr vs. Δ*dIlp^7^*: *p* = 0.0132^∗^; Cntr vs. Δ*dIlp^2–3^*: *p* > 0.9999^*ns*^; Cntr vs. Δ*dIlp^2,7^*: *p* = 0.013^∗∗^. For crude data, see Supporting informations_Raw data_Main figures/Supplementary Figure 3B. (C) Significance calculated by Kruskal-Wallis test, Cntr vs. Δ*dIlp^2^*: *p* = 0.2396^*ns*^; Cntr vs. Δ*dIlp^7^*: *p* = 0.0016^∗∗^; Cntr vs. Δ*dIlp^2–3^*: *p* > 0.9999^*ns*^; Cntr vs. Δ*dIlp^2,7^*: *p* = 0.0012^∗∗^. For crude data, see Supporting informations_Raw data_Main figures/Supplementary Figure 3C. (D) Starved flies were transferred onto plates loaded with protein baits at 20°C and hemolymph was taken after set time intervals (in hours, h; 0, 1, and 2 h). Plotted are measured hemolymph protein levels of genetic controls (*mCherry:FOXO*, Cntr) and different dIlp mutants as indicated. Error bars represent standard deviation; plotted are the mean. Significances calculated by Dunnett’s multiple comparison test but not shown to avoid overloaded graph, 0 h: Cntr vs. Δ*dIlp^2^*: *p* = 0.072^∗∗^; Cntr vs. Δ*dIlp^7^*: *p* = 0.9132^*ns*^; Cntr vs. Δ*dIlp^2–3^*: *p* = 0.4179^*ns*^; Cntr vs. Δ*dIlp^2,7^*: *p* = 0.8744^*ns*^. 1 h: Cntr vs. Δ*dIlp^2^*: *p* = 0.9990^*ns*^; Cntr vs. Δ*dIlp^7^*: *p* = 0.4465 ns; Cntr vs. Δ*dIlp^2–3^*: *p* = 0.0002^∗∗∗^; Cntr vs. Δ*dIlp^2,7^*: *p* = 0.8939^*ns*^. 2 h: Cntr vs. Δ*dIlp^2^*: *p* < 0.0001^****^; Cntr vs. Δ*dIlp^7^*: *p* = 0.0260^∗^; Cntr vs. Δ*dIlp^2–3^*: *p* = 0.0285^∗^; Cntr vs. Δ*dIlp^2,7^*: *p* = 0.047^∗∗^. For crude data, see Supporting informations_Raw data_Main figures/Supplementary Figure 3D.

It remains unclear how dIlp2 and dIlp7 regulate metabolism in larval development, and if wild larvae are exposed to yeast-free food sources. In contrast to larvae, wild adult flies feed partly on yeast free diets (Knittelfelder et al., 2020). To test if dIlp2 and dIlp7 are essential for adult survival, we kept Δ*dIlp^2^*, Δ*dIlp^3^*, Δ*dIlp^7^*, Δ*dIlp^2,3^*, and Δ*dIlp^2,7^* animals on CF. Contrary to our larval data, except for Δ*dIlp^3^*, all other tested mutants show lower survival rates with respect to genetic controls (Figure 3A). Induced starvation could represent one simple explanation to our findings. To investigate feeding behavior, we colored the food with Bromophenol-blue and inspected the digestive tract of feeding flies after a set time-interval. Like reported (Cognigni et al., 2011; Semaniuk et al., 2018), we found that Δ*dIlp^2^*, Δ*dIlp*^7^, and Δ*dIlp^2,7^* flies ingest food faster than controls (Figure 3B). Moreover, we confirmed that the transition of ingested material takes longer in these three mutants (Figure 3C). However, our assays do not assess the capability of nutrients to pass the gut-blood barrier. To assess the problem, we have established a protein-tolerance test. We starved animals for a periode of time, subsequently fed the flies with soy-peptone and measured changes of the hemolymph protein concentration. We found that Δ*dIlp^2^*, Δ*dIlp^7^*, and Δ*dIlp^2,3^* absorb dietary proteins at slower rates than genetic controls (Figure 3D). Interestingly, starved Δ*dIlp^2,7^* flies have high protein levels in circulation with respect to controls and keep their hemolymph protein yields remarkably constant for the time of the experiment (Figure 3D). Taken together, on CF, dIlp7 is required in adults to regulate food ingestion and absorption across the gut-blood barrier.

### Dilp7 Regulates Systemic Insulin Signaling in Animals Kept on Corn Food

**FIGURE 4:**
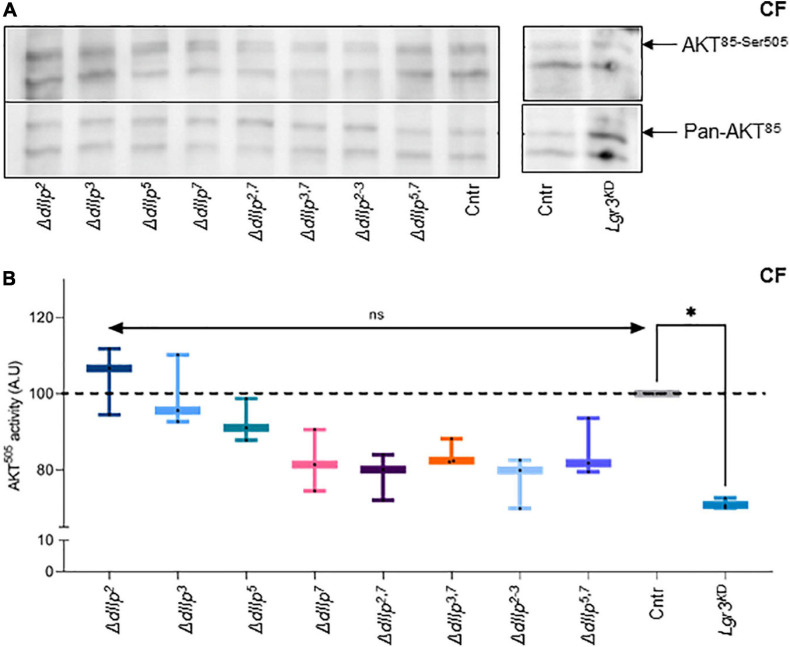
DIlp7 regulates systemic Insulin signaling in adult fed on yeast-free plant diet. **(A,B)** Adult head samples were probed with antibodies for phosphorylated AKT^85–Ser505^ and total AKT amounts (pan-AKT). Shown are western blot photographs from genetic control (*mCherry:FOXO*, Cntr) and different dIlp mutants kept on corn food (CF) at 20°C. AKT^85–Ser505^ / pan-AKT signal ratios were normalized to controls and plotted as AKT^505^ activity **(B)**. Genetic controls (*mCherry:FOXO*, Cntr), different dIlp mutants and flies with Lgr3 knocked-down in IPCs (*Lgr3*^*KD*^) were measured and plotted on box-plots. The activity of each genotype was normalized to the control (*Cntr*, gray, median = 100%). Significances not shown if the n is not enough significant (*n* < 3). Statistics, Kruskal-Wallis test, *p* > 0.05^*ns*^; *p* = 0.0145^∗^. For crude data, see Supporting informations_Raw data_Main figures/Supplementary Figure 4B.

Food ingestion and intestinal transition time are critical for efficient absorption of nutrients. DIlp2 and dIlp3 are known to regulate cellular uptake of macronutrients from circulation (Semaniuk et al., 2018). To investigate if dIlp7 is involved in the regulation of the cellular IS cascade activity on yeast free diet, we quantified the phosphorylation state of AKT in different mutant backgrounds. In adult head samples, two AKT isoforms are detectable, and the phosphorylation state of the isoform AKT^85^ is instructive for sugar uptake into cells (Trautenberg et al., 2019). Moreover, AKT possesses multiple phosphorylation sites and antibodies are in exsitance to detect phosphorylation at AKT^*Thr–342*^ and AKT^*Ser–505*^ (Trautenberg et al., 2019). We could not detect differences in the AKT^85–Thr342^ phosphorylation state for any of the tested genotypes kept on CF (Supplementary Figure 3A). This also seems to be true for flies kept on YF (Supplementary Figure 3B). In contrast, we found AKT^85–Ser505^ phosphorylation reduced in Δ*dIlp^7^*, Δ*dIlp^2–3^*, and all tested dIlp-Δ*dIlp^7^* double mutants (Figures 4A,B). To investigate, if diet can compensate for the loss of AKT activity, we repeated the experiments performed on CF with YF. Our data suggest that in all tested genotypes the AKT phosphorylation levels do not deviate strongly from controls (Supplementary Figures 3C,D).

We speculated that on yeast-free food, dIlp7 regulates IPC activity by activating one of its three predicted candidate receptors: the *Drosophila* Insulin-like receptor (dInR) (Brogiolo et al., 2001), or one of the two predicted G-protein coupled receptors (GPCRs), Leucine-rich repeat-containing G protein-coupled receptor 3 (Lgr3) and 4 (Lgr4) (Veenstra et al., 2012; Colombani et al., 2015; Garelli et al., 2015; Veenstra, 2016). We decided not to target InR, since it was shown that an dInR knock-down in IPCs results in the reduction of dIlp2 (Nässel et al., 2013). However, our findings with dIlp2 mutants on CF did not show any reduction of AKT activity. Lgr4 shows a strong sex specific expression in larval and adult brains (Van Hiel et al., 2015). Thus, we ruled Lgr4 as an unlikely candidate to convey signals between D7Ns and IPCs (Supplementary Figure 3D). To test if Lgr3 is targeted by dIlp7 in adult flies, we knocked down this receptor by RNA interference (RNAi) in IPCs using published validated genetic tools (Jaszczak et al., 2016). The knock down of Lgr3 (Lgr3^*KD*^) in IPCs of adult flies kept on CF reduced AKT^85–Ser–505^ phosphorylation to levels assimilable to tested Δ*dIlp^7^* mutant backgrounds (Figures 4A,B). In contrast, the genetic reduction of Lgr3 in siblings kept on YF did not show any activity change of AKT^85^ (Supplementary Figures 3C,D). Taken together, we conclude that YF can compensate for the loss of dIlp7 in D7Ns or Lgr3 by IPCs. However, on yeast-free diet, adult flies rely on dIlp7 signaling to adjust their feeding behavior and to maintain basic AKT activity.

## Discussion

We have analyzed the role of dIlp7-producing neurons in different thermal treatments. D7Ns are active on yeast diets (Linneweber et al., 2014), but show no activity in animals kept on yeast-free corn food (CF). We found that activated D7Ns are required to respond to heat stress. In addition, we show that dIlp7 produced by D7Ns regulates dIlp2/dIlp3-induced Insulin signaling (IS) on CF, and that yeast products are able to supplement efficiently for the loss of this neuropeptide.

The generative cycle of *Drosophila* is divided into feeding and non-feeding stages. Due to the absence of food intake during embryonic and pupal development these stages highly rely on internal energy stores. In contrast, larvae and adults need to absorb food to survive and develop. The IS cascade is one metabolic circuit to regulate the absorption and internal turnover of macronutrients. In addition, the IS is essential to provide thermal resistance for ectothermic insects (Hamada et al., 2008; Li and Gong, 2015; Umezaki et al., 2018). All feeding stages of *Drosophila* express four neuronal Insulin-like peptides, namely dIlp 2, 3, 5, and 7 (Brogiolo et al., 2001; Rulifson et al., 2002; Grönke et al., 2010). Larvae with functionally compromised Insulin-producing cells (IPCs) kept on yeast diets are heat sensitive, slow in development and small in size (Rulifson et al., 2002; Géminard et al., 2006; Boulan et al., 2015; Li and Gong, 2015; Brankatschk et al., 2018).

Dietary yeast increase intracellular Ca^2+^ levels of IPCs, elevate systemic IS and support survival at high temperatures (Brankatschk et al., 2014, 2018). We found that IPCs with high Ca^2+^ are not sufficient to rescue larval survival at high temperatures on yeast-free CF. Therefore, we speculated that yeast products likely activate additional neurons involved in heat stress responses. It was shown that animals kept on yeast increase Ca^2+^ in D7Ns (Linneweber et al., 2014). D7Ns connect to IPCs and are able to stimulate the latter (Cognigni et al., 2011; Nässel et al., 2013). We show that, on CF, D7Ns are low on Ca^2+^ with respect to yeast-fed animals and that induced Ca^2+^ levels in D7Ns improve larval heat resistance on CF. In addition, larvae with inactivated D7Ns kept on yeast show poor survival at high temperatures. Thus, D7Ns are one integral part of the heat response and we speculate that these neurons directly communicate with IPCs. D7Ns secrete a multitude of neuropeptides including dIlp7 (Miguel-Aliaga et al., 2008; Nässel et al., 2008; Cognigni et al., 2011; Carlsson et al., 2013). DIlp7 mutants kept on yeast food (YF) are slightly heat sensitive, and due to such relative high survival rates, we deem it unlikely that dIlp7 is one main cue crucial to withstand thermal treatments.

D7Ns are inactive on CF and we hoped to identify dIlp candidates responsible for IS on yeast-free diets. Interestingly, we identified dIlp2, dIlp3, and dIlp7 essential for larval development. Moreover, genetic interactions revealed that Δ*dIlp^2,3^* double mutants are unable to survive on CF. In stark contrast, Δ*dilp^2,7^* and Δ*dilp^2–3,7^* animals rescued the lethality shown by single mutants. Our findings indicate a new metabolic link between dIlp7 and dIlp2 essential for larval development in yeast-free environments. However, wild larvae grow in microbe-rich environments, such as rotting fruits, and have likely access to dietary yeast. Adult flies sometimes feed on yeast-poor diets or avoid yeast in response to cold (Brankatschk et al., 2018; Knittelfelder et al., 2020). Therefore, we decided to sample adults kept on CF. We found that adult Δ*dilp^7^* flies show reduced IS levels and higher lethality rates with respect to genetic controls. Moreover, the combined absence of dIlp2 and dIlp7 pronounced the observed adult lethality on CF. Thus, larval and adult dIlp7 signaling is likely very different.

It was reported that dIlp7 is expressed in the subesophageal ganglion region of the brain and suggested that D7Ns regulate the feeding behavior (Cognigni et al., 2011). Therefore, reduced feeding of dIlp7 mutants could explain the lower IS levels on CF. We show that, on CF, Δ*dilp^2^*, and Δ*dilp^7^* mutants ingest food faster, have a longer retention time of the ingested material and are able to absorb macronutrients. Therefore, we do not favor the idea that these flies are starving on CF. It is more likely that dIlp7 is required to stimulate IPCs to maintain basic dIlp levels in circulation. To test for this possibility, we decided to knock down the predicted target receptor of dIlp7, the G-protein-coupled rector Lgr3 (Veenstra et al., 2012; Colombani et al., 2015; Garelli et al., 2015; Veenstra, 2016). We found that the loss of Lgr3 results in low IS levels on CF. In contrast, on YF, all tested genotypes show IS comparable to controls. Taken together, we conclude that neuronal dIlp7/Lgr3 signaling controls IPCs in adults kept on yeast-free diets. As such dIlp7 secures a basic amount of systemic IS and therefore, likely contributes to thermal resistance of adult flies. However, required adult tracking on CF at low temperatures appeared impractical to confirm our idea (Brankatschk et al., 2018).

Neuronal innervation of IPCs is established in many animals and modulates metabolic signals (Rodriguez-Diaz et al., 2011; Taborsky, 2011; Nässel et al., 2013; Li and Gong, 2015). Our findings indicate that food products can overwrite such neuronal stimulation. In *Drosophila*, we found a dual role for D7Ns: (i) these neurons facilitate the heat response of larvae feeding on yeast and (ii) they form a metabolic circuit that enables adult flies to thrive on yeast-free diets if required. In mice and humans, pancreatic islets are directly innervated (Rodriguez-Diaz et al., 2011; Taborsky, 2011); however, the role of this neuronal stimulation in response to dietary cues is not well understood. We have identified the importance of D7Ns and their product, dIlp7, to regulate IS in response to dietary quality. Our findings provide new insights into the neuronal stimulation of IPCs within a given ecological context and provide a model to study neuronal innervation of insulin producing cells.

## Materials and Methods

### Stocks

If not stated, stocks were raised on normal food at 20–22°C (Table 3).

**TABLE 3:** Normal food recipe.

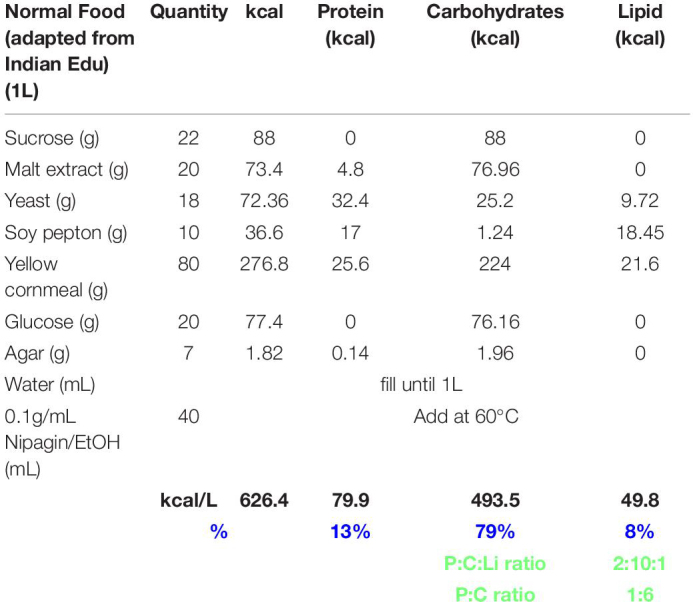

*Recipe for 1liter of normal food. The calories (in kcal) brought by each ingredients have been calculated. At the bottom, in black bold, the totals (in kcal) aer shown. In blue are indicated the proportions (%) of kcal of proteins, carbohydrates, or lipids on the total amount of calories. In green are shown the protein: carbohydrate: lipid and protein: carbohydrate ratios. EtOH, ethanol; P, proteins; C, carbohydrates; Li, lipids.*

The following lines were used:

*w;foxo[mCherry]* (#80565) were provided by Bloomington Drosophila Stock Center (BDRC). *W*^1118^ were provided by S. Eaton’s laboratory.

*Drosophila* insulin-like peptide mutants: *w*^∗^; *dilp1^1^- w*^∗^; *dilp5^1^, yw; dilp6^1^, w*^∗^; *dilp7^1^, w*^∗^; *dilp2-3^1^* and *w*^∗^; *dilp2-3,7*^1^ ((Grönke et al., 2010); from S. Grönke); *w*^∗^; Δ*dilp^2–3,5^ /TM3,GFP* from R. Kühnlein.

Δ*dIlp* mutants were backcrossed for five generations into *white*^1118^ stocks.

Gal4 lines: *w;dilp2-Gal4* (Rulifson et al., 2002); #37516 from BDRC); *w;dilp7-Gal4* [(Cognigni et al., 2011); gift from I. Miguel-Aliaga].

UAS-lines: *w;UAS-TrpA1* (#26264, from BDRC); *w;UAS-Kir2.1-egfp,tub80ts* (from S. Eaton); *w;UAS-GcAMP* (#32116 from BDRC); *w;UAS-Lgr3shRNA* (v330603; from VDRC)**;** *w;UAS-Lgr4RNAi* (v102681; from VDRC).

### Diets

Normal food was adapted from https://bdsc.indiana.edu/information/recipes/bloomfood.htm.l (Table 3). Yeast food was prepared as published by Brankatschk et al. (2018) (Table 1). Corn food recipe is described in Table 2 and in Trautenberg et al. (2020).

### Development Tracking Experiments

#### Experimental Settings

Embryos were collected overnight on apple-juice agar plates [25% apple-juice, 1.5% agar, 0.4% p-Hydroxybenzoic acid methyl ester (nipagin)], bleached in 80% bleach/water for 30 s, rinsed and kept for 24 h on starvation plates (25% apple juice, 10% Glucose, 1.5% agar, 0.4% nipagin) at 20–22°C. First-instar larvae were pipetted in 10 μL 0.00005% Triton-X100/PBS solution and transferred in 96 well-plates containing food. One larva per well (containing 200 μL of food) was deposited. Later, the plates were sealed with a poked ventilated plastic film and put in incubators set at the temperature of interest. Both developmental speed/rate (how many days each larva took to reach the pupal stage) and success (how many larvae succeed to reach the pupal stage on the total number of larvae plated on one plate).

Kir2.1 induction at different stages: The egg progeny of crosses between *UAS-Kir2.1, tub80ts* or *white*^1118^ with *dilp2-Gal4* were collected. Larvae and plates were prepared as described above. Plates were kept at 20°C for 24, 72, or 96 h and then transferred to the 28°C-incubator.

### Statistics

To calculate the success rates, only plates with a minimum total of 10 individual larvae for 20°C or six individual larve for 28°C/induction experiments, were taken into account. For developmental success and speed, data points outside the range of the first standard deviation (i.e., Mean + or − Standard deviation) were excluded. Mann-Whitney or Kruskal-Wallis tests were performed as statistical tests (PRISM Graphpad software).

### Immunohistochemistry

The larvae were dissected, turned as socks in fix solution and kept in fix solution for 20 min. The carcasses were stained by HrpCy5 (HRP-S5-1; NANOCS). The central nervous systems were dissected from the carcasses and mounted in 50% Glycerol/PBS. Confocal microscopy was performed on a Zeiss confocal laser scanning microscope LSM 700 of the Light Microscopy Facility, a Core Facility of the CMCB Technology Platform at TU Dresden. Antibody used: anti-dIlp7 [gift from Irene Miguel-Aligua (Cognigni et al., 2011)].

### GcaMP Experiments

#### Sample Collection

The egg progeny of crosses between UAS-GCaMP or Canton S and *dilp2-Gal4* or *dilp7-Gal4* were collected. Larvae were prepared as described above in “developmental tracking part.” Three larvae were pipetted in 10 μL 0.00005% Triton-X100/PBS solution and transferred in 24 well-plates containing food. Three larvae per well (containing 1 mL of food) were deposited. Larvae were kept on either YF or CF and staged specimen were collected respective to their biological age.

For GcaMP detection, the samples were prepared as previously described by Brankatschk et al., 2014 (Brankatschk et al., 2014). The larvae were dissected in ice cold fix solution (4% Paraformaldehyde in Graces medium) and fixed at RT for 20 min. The brains were mounted in Vectashield. Confocal microscopy was performed on a Zeiss confocal laser scanning microscope LSM 700 of the Light Microscopy Facility, a Core Facility of the CMCB Technology Platform at TU Dresden.

#### Quantification

The total number of dilp2- or dilp7-GcaMP-postive (i.e., green-fluorescent cells) were counted in each brain imaged. The percentage of cells ON was then calculated on the total number of dilp2-positive cells (i.e., 8 per hemisphere (Rulifson et al., 2002) or dIlp7-positive cells [i.e., 12 (Miguel-Aliaga et al., 2008)] Statistics, Mann-Whitney’s test.

### Pupal Area

Pupae arising from larvae reared in 96-well plates containing yeast food at 20°C were placed on microscope slides were a 5 mm-scale bars were drawn. Pictures were taken with a camera and processed by using Fiji (Schindelin et al., 2012). By using the “freehand” tool, the area of each pupa was measured. Mann-Whitney’s tests were performed as statistical analysis.

### Feeding Behavior

#### Adult Food Intake

Seven-to-ten days old adults fed on corn food for 48 h were transferred in vials containing 5 mL 0.5% Bromophenol-Blue colored corn food (Cognigni et al., 2011). The male: female ratio per vial was 5:15. After 4 h at 20°C, the number of females with blue guts were counted. Finally, the percentage of females with blue guts after 4 h on the diet was plotted. Statistics, experiments out of the range (i.e., Mean + or – Standard deviation) were excluded of the normalized data. Kruskal-Wallis tests were performed as statistical tests.

#### Adult Intestinal Food Transit

Flies used for the food intake experiments (see above), kept for 4 h into vials containing 5mL 0.5% Bromophenol-Blue colored corn food (Cognigni et al., 2011) were transferred into vials containing uncolored corn food. The male: female ratio per vial was 5:15. After 4 h at 20°C, the number of females with blue guts were counted. Finally, the percentage of females with blue guts after 4 h on the diet was plotted. Statistics, experiments out of the range (i.e., Mean + or – Standard deviation) were excluded of the normalized data. Kruskal-Wallis tests were performed as statistical tests.

### Biochemistry

#### AKT Detection in Yeast and Corn Food-Fed Adult Heads

Sample preparation, western-blotting and quantification were performed as published by Trautenberg et al. (2019). In brief, flies were raised on normal food and 5 days-old adults were transferred onto respective experimental foods for 7 days at 20°C. Thereafter, flies were snap-frozen in liquid nitrogen and frozen heads from females collected for processing. Heads were homogenized on ice in 0.01% Tx100-PBS and subsequently cooked at 95°C for 5 min. Polyclonal antibodies used to probe were Akt-pSer505 (Cell Signaling, 4054S), Akt-pThr308 (Invitrogen, 44-602G), Akt (Invitrogen, MAS14916).

#### Quantification

Pixel intensity of defined area (line) covering the signature (not saturated) center on photograph was measured using FIJI software (Schindelin et al., 2012). Intensity ratio between AKT85-Ser505 and panAKT85 was calculated and normalized to value obtained from controls.

### Protein Quantification in Adult Hemolymph

#### Sample Collection and Preparation

Eight-to-ten days-old adults were starved (25% apple juice, 10% Glucose, 1.5% agar, 0.4% nipagin) at 20–22°C for 18 h. Soy pepton solution was then added on the plate and flies were collected at time 0 (when the protein solution is added), 1 and 2 h after soy pepton solution added. Flies were transferred into 2 mL Eppendorf tubes and short frozen in liquid nitrogen. To collect hemolymph, 110 μL of ice-cold 1xPBS was added to a tube containing sixteen adults (male: female ratio ≈ 4:12). Samples were incubated on ice for 5 min and centrifuged for 10 s (benchtop centrifuge) two times, then 100 μl of the supernatant was transferred to a fresh tube.

#### Protein Assay and Quantification

Proteins in hemolymph samples were concentrated by choroform-methanol precipitation and re-dissolved in 0.1% TritonX100 in 1xPBS. Protein amount was measured and quantified like recommended by manufacturer (Pierce^TM^ BCA Protein Assay Kit, Thermo Fisher Scientific).

## Data Availability Statement

The original contributions presented in the study are included in the article/Supplementary Material, further inquiries can be directed to the corresponding author.

## Author Contributions

MB and EP: conceptualization and project administration. EP: formal analysis, validation, and visualization. MB: funding acquisition. EP, JK, LT, and SB: investigation. EP and MB: methodology, resources, and supervision. MB, EP, JK, and LT: writing – original draft preparation. All authors contributed to the article and approved the submitted version.

## Conflict of Interest

The authors declare that the research was conducted in the absence of any commercial or financial relationships that could be construed as a potential conflict of interest.

## Publisher’s Note

All claims expressed in this article are solely those of the authors and do not necessarily represent those of their affiliated organizations, or those of the publisher, the editors and the reviewers. Any product that may be evaluated in this article, or claim that may be made by its manufacturer, is not guaranteed or endorsed by the publisher.

## Supplementary Material

Supplementary Figure 1 Larval heat resistance is dependent on dietary cues and activated IPCs. **(A)** Larvae kept on yeast food (YF) were exposed to heat stress at different developmental stages. Shown is the larval survival (percentage of pupariation) of controls (Cntrs; pool of *dilp2Gal4/+* and *UAS-Kir2.1/+* percentages of pupariation) and larvae with silenced IPCs (*dilp2 > > Kir2.1*). Each dot represents one experiment with minimum *n* = 6 individual larvae, experimental time axis indicated (top). Significance calculated by Mann-Whitney’s test (from left to right), *p* < 0.9999^*ns*^, *p* = 0.0048^∗∗^, *p* = 0.0040^∗∗^, *p* = 0.0079^∗∗^, *p* = 0.0571^*ns*^. For crude data, see Supporting informations_Raw data_Supplementary Figure 1A. **(B)** Survival of larvae kept on corn food (CF) at different temperatures (20° or 28°C). Shown is the larval survival (percentage pupariation) of controls (Cntrs; pool of *dilp2Gal4/+* and *UAS-TRPA1/+* percentages of pupariation) and larvae with activated IPCs (*dilp2 > > TRPA1*). Each dot represents one experiment with minimum *n* = 6 individual larvae, experimental time axis indicated (top). Significance calculated by Mann-Whitney’s test, *p* > 0.05. For crude data, see Supporting informations_Raw data_Supplementary Figure 1B. **(C)** Confocal imaging of dIlp7-neurons expressing GCaMP (green) and CherryNLS (magenta) in third-instar larval brains (kept on food with yeasts). The photographs C.a. – C.b. depict fixed samples and the photographs A.d. are from life-recordings. Shown are maximum intensity projections (2 μm z-stack thickness). Scale bar = 100 μm. **(D)** Plotted are survival rates of controls (Cntr, *mCheery:FOXO*) and Δ*dIlp^7^* mutants kept on yeast food (YF) at 20°C. Each dot represents one experiment with minimum *n* = 10 individual larvae, experimental time axis indicated (top). Significance calculated by Mann-Whitney’s test, *p* = 0.0112^∗^. For crude data, see Supporting informations_Raw data_Supplementary Figure 1D.

Supplementary Figure 2 Larval development depends on IS and food quality. **(A)** Shown are survival rates (percentage of pupariation) of larvae kept on corn food (CF) at 20°C. Plotted are the percentages of pupariated larvae of controls (Cntr, *mCheery:FOXO*) and different dIlp mutants (indicated on the *X*-axis). Each dot represents one experiment with minimum *n* = 10 individual larvae, experimental time axis indicated (top). For crude data, see Supporting informations_Raw data_Supplementary Figure 2A. (B) Shown are survival rates of larvae kept on yeast food (YF) at 20°C. Plotted is the percentage of pupariated larvae of controls (Cntr, *mCheery:FOXO*) and different dIlp mutants (indicated on the *X*-axis). Each dot represents one experiment with minimum *n* = 10 individual larvae, experimental time axis indicated (top). Significance calculated by Mann-Whitney’s test, *p* > 0.05^*ns*^; *p* < 0.01^∗∗^; *p* < 0.001^∗∗∗^. For crude data, see Supporting informations_Raw data_Supplementary Figure 2B. **(C)** Shown are photographs (a–f) of pupae formed from larvae kept on yeast food (YF) at 20°C. Pupal areas (indicated in a., broken line) of genetic controls (Cntr, *mCheery:FOXO*) and different dIlp mutants were measured and plotted in g. Each dot on the box-plot represents one pupa. Significance calculated by Mann-Whitney’s test, *p* > 0.05^*ns*^; *p* < 0.01^∗∗^; *p* < 0.0001^****^. For crude data, see Supporting informations_Raw data_Supplementary Figure 2C.

Supplementary Figure 3 AKT^85–Thr342^ phosphorylation is not dependent from dietary cues. **(A–D)** Shown are quantifications from western blot data probed with specific AKT antibodies. Head samples from adult female flies kept at 20°C on either corn food (CF; **A**) or yeast food (YF; **B–D**) were probed with anti-P-AKT^*Thr342*^ **(A,B)**, anti-P-AKT^*Ser505*^ **(C,D)**, and anti-AKT **(C,D)**. Anti-P-AKT/AKT signal ratios from different dIlp mutants were normalized to controls (Cntr; *mCheery:FOXO*). Each dot on the box-plots represents one data point. Significances not calculated are due to low sample number (*n* < 3). Statistics, Kruskal-Wallis test, *p* > 0.9999^*ns*^ **(A)**; *p* > 0.05^*ns*^ **(B,C)**.
