# Supplementary figures and images for: DIlp7-Producing Neurons Regulate Insulin-Producing Cells in Drosophila
Source: Front Physiol. 2021 Jul 27;12:630390. doi: 10.3389/fphys.2021.630390 (PMC8353279; doi:10.3389/fphys.2021.630390)

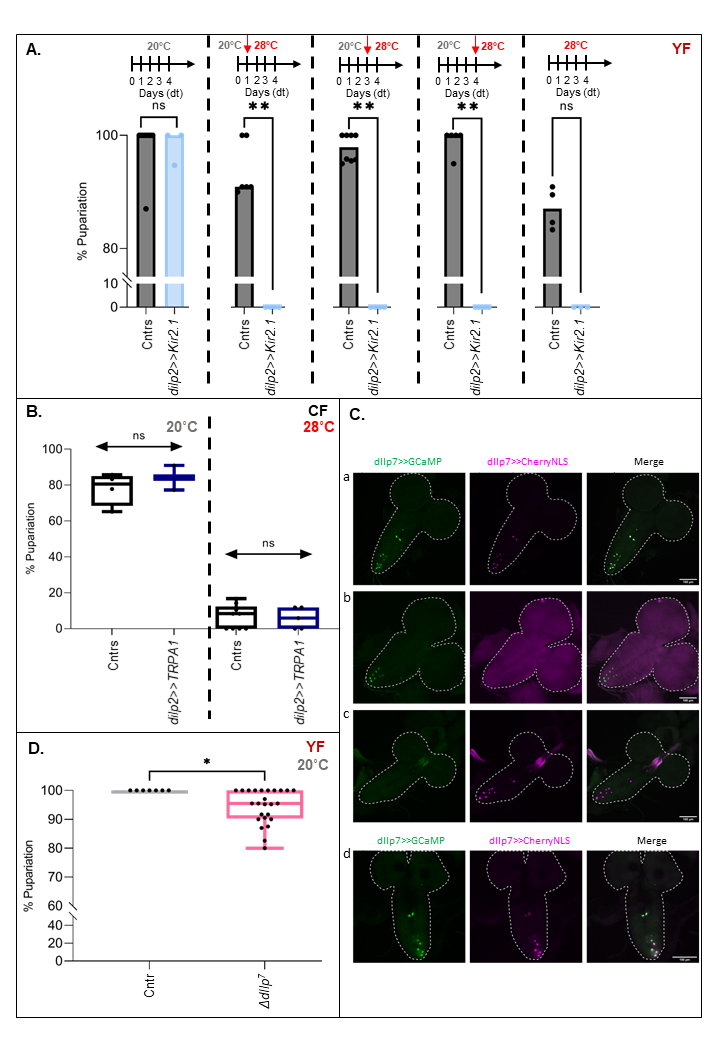

Supplement: Supplementary Figure 1 — Larval heat resistance is dependent on dietary cues and activated IPCs. (A) Larvae kept on yeast food (YF) were exposed to heat stress at different developmental stages. Shown is the larval survival (percentage of pupariation) of controls (Cntrs; pool of dilp2Gal4/+ and UAS-Kir2.1/+ percentages of pupariation) and larvae with silenced IPCs (dilp2 > > Kir2.1). Each dot represents one experiment with minimum n = 6 individual larvae, experimental time axis indicated (top). Significance calculated by Mann-Whitney’s test (from left to right), p < 0.9999ns, p = 0.0048∗∗, p = 0.0040∗∗, p = 0.0079∗∗, p = 0.0571ns. For crude data, see Supporting informations_Raw data_Supplementary Figure 1A. (B) Survival of larvae kept on corn food (CF) at different temperatures (20° or 28°C). Shown is the larval survival (percentage pupariation) of controls (Cntrs; pool of dilp2Gal4/+ and UAS-TRPA1/+ percentages of pupariation) and larvae with activated IPCs (dilp2 > > TRPA1). Each dot represents one experiment with minimum n = 6 individual larvae, experimental time axis indicated (top). Significance calculated by Mann-Whitney’s test, p > 0.05. For crude data, see Supporting informations_Raw data_Supplementary Figure 1B. (C) Confocal imaging of dIlp7-neurons expressing GCaMP (green) and CherryNLS (magenta) in third-instar larval brains (kept on food with yeasts). The photographs C.a. – C.b. depict fixed samples and the photographs A.d. are from life-recordings. Shown are maximum intensity projections (2 μm z-stack thickness). Scale bar = 100 μm. (D) Plotted are survival rates of controls (Cntr, mCheery:FOXO) and ΔdIlp7 mutants kept on yeast food (YF) at 20°C. Each dot represents one experiment with minimum n = 10 individual larvae, experimental time axis indicated (top). Significance calculated by Mann-Whitney’s test, p = 0.0112∗. For crude data, see Supporting informations_Raw data_Supplementary Figure 1D. [file Image_1.tif]

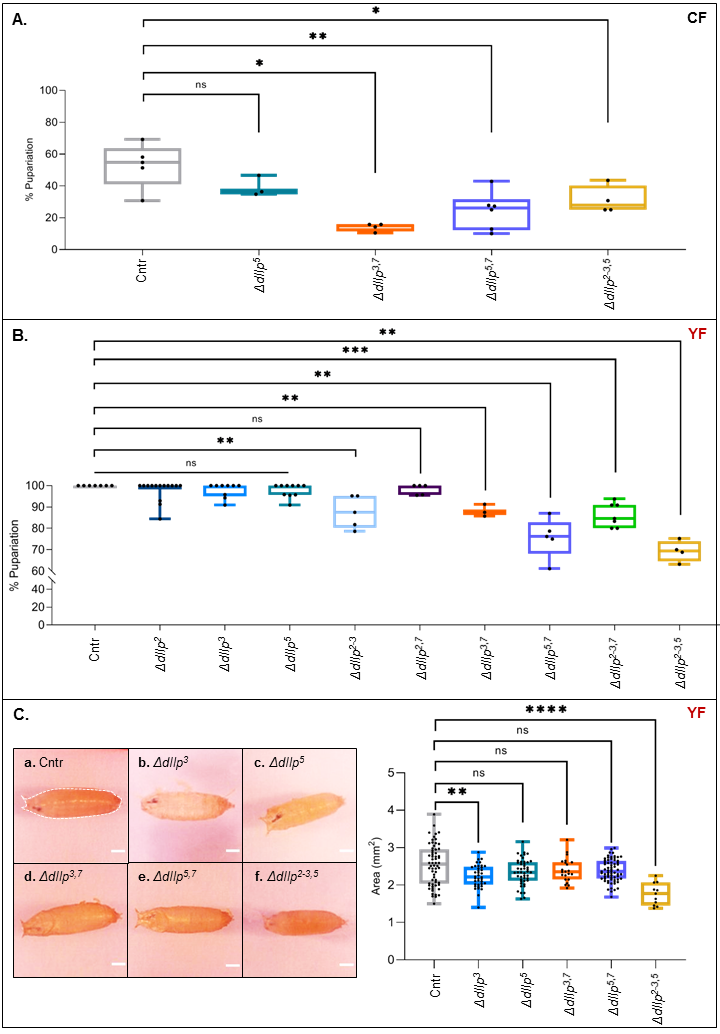

Supplement: Supplementary Figure 2 — Larval development depends on IS and food quality. (A) Shown are survival rates (percentage of pupariation) of larvae kept on corn food (CF) at 20°C. Plotted are the percentages of pupariated larvae of controls (Cntr, mCheery:FOXO) and different dIlp mutants (indicated on the X-axis). Each dot represents one experiment with minimum n = 10 individual larvae, experimental time axis indicated (top). For crude data, see Supporting informations_Raw data_Supplementary Figure 2A. (B) Shown are survival rates of larvae kept on yeast food (YF) at 20°C. Plotted is the percentage of pupariated larvae of controls (Cntr, mCheery:FOXO) and different dIlp mutants (indicated on the X-axis). Each dot represents one experiment with minimum n = 10 individual larvae, experimental time axis indicated (top). Significance calculated by Mann-Whitney’s test, p > 0.05ns; p < 0.01∗∗; p < 0.001∗∗∗. For crude data, see Supporting informations_Raw data_Supplementary Figure 2B. (C) Shown are photographs (a–f) of pupae formed from larvae kept on yeast food (YF) at 20°C. Pupal areas (indicated in a., broken line) of genetic controls (Cntr, mCheery:FOXO) and different dIlp mutants were measured and plotted in g. Each dot on the box-plot represents one pupa. Significance calculated by Mann-Whitney’s test, p > 0.05ns; p < 0.01∗∗; p < 0.0001****. For crude data, see Supporting informations_Raw data_Supplementary Figure 2C. [file Image_2.tif]

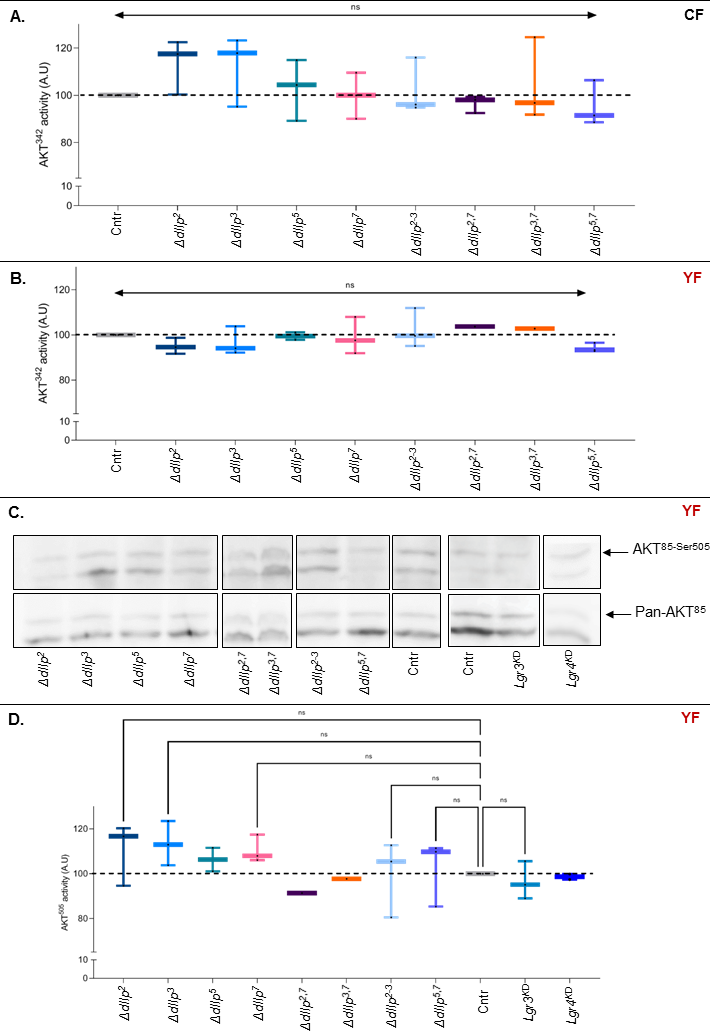

Supplement: Supplementary Figure 3 — AKT85–Thr342 phosphorylation is not dependent from dietary cues. (A–D) Shown are quantifications from western blot data probed with specific AKT antibodies. Head samples from adult female flies kept at 20°C on either corn food (CF; A) or yeast food (YF; B–D) were probed with anti-P-AKTThr342 (A,B), anti-P-AKTSer505 (C,D), and anti-AKT (C,D). Anti-P-AKT/AKT signal ratios from different dIlp mutants were normalized to controls (Cntr; mCheery:FOXO). Each dot on the box-plots represents one data point. Significances not calculated are due to low sample number (n < 3). Statistics, Kruskal-Wallis test, p > 0.9999ns (A); p > 0.05ns (B,C). [file Image_3.tif]
